# The Design of New Adjuvants for Mucosal Immunity to *Neisseria meningitidis* B in Nasally Primed Neonatal Mice for Adult Immune Response

**DOI:** 10.1100/2012/292073

**Published:** 2012-04-01

**Authors:** Tatiane Ferreira, Elizabeth De Gaspari

**Affiliations:** Immunology Department, Adolfo Lutz Institute, Avenue Dr. Arnaldo 355, 11 andar, 01246-902 São Paulo, SP, Brazil

## Abstract

The aim of this study was to determine the value of detoxified Shiga toxins Stx1 and Stx2 (toxoids of *Escherichia coli*) as mucosal adjuvants in neonatal mice for immunogenicity against the outer membrane proteins (OMPs) of *Neisseria meningitidis* B. Mucosal immunization has been shown to be effective for the induction of antigen-specific immune responses in both the systemic and mucosal compartments. Systemic antibody levels (IgG, IgG1, IgG2a, IgG2b, IgM, and IgA) and mucosal IgM and IgA were measured by ELISA using an *N. meningitidis* as an antigen. In addition, IFN-**γ** and IL-6 production were measured after stimulated proliferation of immune cells. Intranasal administration elicited a higher anti-OMP IgA response in both saliva and vaginal fluids. Our results suggest that both Stx1 and Stx2 toxoids are effective mucosal adjuvants for the induction of Ag-specific IgG, IgM, and IgA antibodies. The toxoids significantly enhanced the IgG and IgM response against OMPs with a potency equivalent to CT, with the response being characterized by both IgG1 and IgG2a isotypes, and increased IFN-gamma production. Additionally, bactericidal activity was induced with IgG and IgM antibodies of high avidity. These results support the use of the new toxoids as potent inducing adjuvants that are particularly suitable for mucosal immunization.

## 1. Introduction

The development of efficient and safe adjuvants for use in human vaccines remains both a challenge and a necessity [1]. As the most widely used adjuvants in humans, aluminum salts predominantly induce antibody responses; therefore, discovering new adjuvants is crucial for the development of vaccines that require a cell-mediated response [2, 3].

Although much of the adjuvant research that was carried out in the past can be seen as empirical, the research did sometimes give rise to potent and useful products. Nevertheless, there is a need to develop a new generation of adjuvants that are rationally designed on the basis of recent progress that has been made in our understanding of the immune response, particularly the innate immune response. Additional adjuvant research into the development of effective mucosal vaccines is also necessary to compensate for the often poor immunogenic nature of orally and nasally administered vaccine antigens by instead inducing vaccine antigen-specific humoral and/or cellular immune responses [4].

We know that efforts to develop new strategies to curb global infection in the field are important and currently the development of novel adjuvants that can be nasally or orally administered vaccine antigens to maximize the induction protective antibodies is under investigation in several laboratories. Thus far, several bacterial enterotoxins, including cholera toxin (CT) of *Vibrio cholerae *and heat-labile enterotoxin (LT) of enterotoxigenic *Escherichia coli*, have been identified as possessing strong immunoenhancing activity against coadministered protein antigens when administered by oral or nasal routes [5, 6].

*Neisseria meningitidis* is a major cause of bacterial meningitis in the human population, especially among young children. There is a need to develop a noncapsular vaccine to prevent meningococcal B infections due to the inadequate immune response elicited against the capsular polysaccharide of these strains. A vaccine inducing protection against most of the circulating variants of serogroup B meningococcal strains is not yet available. Several outer membrane protein- (OMP-) based vaccines for group B meningococcal disease have shown 50 to 80% efficacy in older children [7]. However, efficacy in young children receiving the same vaccines was much lower, despite the induction of high levels of antibody [8].

Colonization of the human nasopharyngeal region by *N. meningitidis* is believed to lead to natural immunity. In some cases, this colonization also initiates the pathogenic process that leads to invasive meningococcal disease. Serum bactericidal antibody, which develops after exposure to meningococcal antigens [9, 10], has been correlated with immunity to meningococcal disease, but mucosal immunity at the portal of entry may also play an important role. A number of plausible options are currently under investigation as a mucosal vaccine to this pathogen [11].

Shiga toxin (Stx) was found to possess immunogenicity but not adjuvant activity when given via the oral route [12]. Stx, which is generated by Stx-producing *E. coli *(STEC), is one of the major virulence factors for STEC infectious diseases. Stx is a holotoxin composed of an A subunit measuring approximately 32 kDa in a noncovalent association with a pentameric ring of identical B subunits, each with a molecular mass of 7.7 kDa [13]. Stxs, which are also known as Shiga-like toxins or Vero toxins, are produced by the enteric pathogens *Shigella dysenteriae *and enterohemorrhagic *E. coli *(EHEC).

Stx is classified into two groups: Stx1, the amino acid sequence which is identical to that of Shiga toxin, and Stx2, which is immunologically distinct from Stx1 [14].

In an effort to develop a candidate adjuvant for a vaccine against infectious diseases caused by *N. meningitidis* B, we have assessed the capability of nasally administered detoxified Stx1 or Stx2 toxins (toxoids) with the OMP of *N. meningitidis*. In this study, we used newborn mice (1-2 weeks of age), which reflect the first year of a human infant's immune maturation in many aspects, as a model [15].

This study is preceded by another published work by Ohmura-Hoshino et al. [16] that described the nontoxic Stx derivatives from *E. coli* possessing adjuvant activity for mucosal immunity. Ohmura et al. used ovalbumin as an antigen and Stx1 as an adjuvant for nasal immunization of adult mice.

## 2. Materials and Methods

### 2.1. Bacterial Strains and Antigen Preparation

The Brazilian epidemic group B meningococcal strain (B:4:P1.15,19,5.5,L3,7,9,1,8) was selected for use in this study. The bacteria were grown overnight in a candle jar on Tryptic Soy Broth (TSB; Difco BRL products, Gaithersburg, MD) supplemented with 1% horse serum (Sigma, St. Louis, MO) in plates in a 5% CO_2_ atmosphere at 37°C. The OMP were prepared by extraction of bacteria with 0.5% deoxycholate in 0.1 M Tris-HCl buffer (pH 8.6) containing 10 mM EDTA and purified by differential centrifugation [17].* E. coli* used in these experiments was obtained from clinical samples from the state of Bahia, Brazil, and was designated as (C7-88) O157:H7 (Stx1) and (1189) ONT: H49 *stx2*+*stx2vb*−*hb *(Stx2) [18]. They were kindly provided by Dr. Roxane Piazza (Bacteriology Laboratory—Butantan Institute, São Paulo, Brazil). *E. coli* bacterial strains were grown in Luria-Bertani (LB) medium supplemented with appropriate antibiotics at 37°C for 18 h under constant shaking (200 rpm). The toxins were detoxified as previously described [19]. Bacteria were centrifuged at 5000 x g, and the supernatant was filtered through a 0.45 *μ*m membrane. These conditions were employed in all experiments. The total protein concentration was determined by the Bradford method using reagents from BioRad (Hercules, CA, USA) and serum albumin as the standard [20].

### 2.2. Animal Experiments

For the generation of newborn mice, pregnant females were separated and caged individually, and newborns were maintained with their mothers during the experiments. Groups of BALB/c* H-2^d^* haplotype neonatal mice were immunized 4 times during 12 days (days 3, 7, 9, and 12) by intranasal (i.n.) immunization with 20 *μ*g of *N. meningitidis* OMP and 2 *μ*g of Stx1 or Stx2 toxoids, a new mucosal adjuvant [17], or CT, applying 2.5 *μ*L per nostril. In another set of experiments, the immunization was performed with the same amount of saline plus adjuvants Stx1, Stx2, or CT, or OMP as experimental controls for immunization. The immunogenicity of the different formulations containing OMP and Stx1, Stx2, or CT was assessed using female BALB/c mice. Mice were distributed into four groups. Serum samples were taken at 28 days after i.n. immunization and 42 days after i.m. immunization. On day 42, mice were sacrificed and spleen cells were obtained for cytokine determinations. Serum was obtained from the mice 28 days after i.n. immunization or at 42 days i.m by retro-orbital puncture after i.m. immunization on the 35th day with OMP (20 *μ*g) and 2 *μ*g of Stx1 or Stx2 as adjuvants. Additionally, New Zealand white rabbits (CRIEX Laboratories, São Paulo, SP, Brazil) weighing 2.25 to 3.5 kg were used in all experiments. We used three rabbits for each experimental group. Prime boosters were administered with i.n. and i.m. immunizations in unanesthetized rabbits. Rabbits were held in a supine position and a flexible micropipettor was used to drip 500 *μ*L (50 *μ*g of stx1 or stx2) of toxoid into the naris, with about half the volume in each nari as described previously [21]. All procedures with the animals were in accordance with the guidelines of the Brazilian Code for the Use of Laboratory Animals.

### 2.3. Monoclonal Antibodies

Murine anti-Stx1 and anti-Stx2 mAbs were secreted from hybridomas generated by a fusion between the lymph node cells of BALB/c mice immunized with Stx1 or Stx2 toxoids and *P*3.653 Ag 8 myeloma cells, as described previously [22–25]. To test the action and specificity of these mAbs, the culture supernatant was assayed for the presence of secreted antibodies by capture ELISA using biotinylated isotypes mouse antibodies (Kirkegaard and Perry, Gaithersburg, MD).

### 2.4. Purification of Stx1 and Stx2 Toxoids

The toxoids were purified by affinity chromatography through Sepharose 4B-mAbs for Stx1 or Stx2 produced in our laboratory, as described by the manufacturer. The antigen was eluted with 0.1 M glycine (pH 3.0) and dialyzed in 0.02 M PBS (pH 7.2). The antigen was concentrated by ultra-filtration with a diaflomembrane of PM 10 (MW, cut-off 10,000; Millipore Corp., MA, USA), and purification was verified by SDS-PAGE analysis. These two toxins were made nontoxic by dialyzing them for 7 days at 37°C in a 100 mM sodium phosphate buffer (pH 8.0) containing 0.6% formaldehyde [26]. Detoxification proceeded at 30°C. The formalized toxin was sampled at intervals by mouse inoculation until it became completely nontoxic. At each interval, 2 mice were i.p. administered with 0.5 mL of the sample and then observed for 4 days. The toxoids were shown to be nontoxic when mice neither died with the toxicity nor showed any specific symptoms of toxicity, such as muscle spasms, stiffening, or any other abnormal signs during the observation period. The toxoids were kept at −20°C until use. After purification, endotoxin concentrations in Stx1 and Stx2 toxoids were determined by the *Limulus *assay (BioWhittaker). CT was obtained from Sigma Chemical Co., St. Louis, MO, USA.

### 2.5. SDS-PAGE

It was performed using a 13% gel. [27]. Briefly, 10 *μ*g of OMP or 30 *μ*g of the toxoids Stx1 or Stx2 and CT were applied to a 13% SDS-polyacrylamide gel. After electrophoresis, the separated proteins were transferred to a nitrocellulose membrane (Hybond C-Extra, Amersham Biosciences, Little Chalfont, UK) at 100 V for 18 h at 4°C. The membrane was blocked with 5% skim milk for 2 h and reacted with a 1 : 50 dilution of Stx1 or Stx2 monoclonal or polyclonal antibodies. The membrane was then washed and incubated for 2 h with peroxidase-conjugated goat anti-mouse IgG or IgM (1 : 2000), or goat anti-rabbit IgG(1 : 5000). After washing, the strips were washed, and the reaction was developed with AEC (Pierce Inc, IL, USA) and stopped by adding distilled water.

### 2.6. Antigen Specificity of the Antibody Responses: Immunoblot

The samples were analyzed by immunoblotting. Briefly, 10 *μ*g of OMP or 20 *μ*g of Stx1 or Stx2 preparation was analyzed by 13% SDS-PAGE and transferred onto 10 mm PVDF strips (Pierce Inc, Illinois, USA). These strips were cut and blocked with 5% skim milk in solution for 2 h at room temperature. Samples were diluted 1 : 50 in blocking solution and incubated for 18 h at 4°C. The strips were washed three times in PBS, and peroxidase-conjugated anti-y, -*μ*, or -*λ* chains, which had been diluted in PBS plus 2.5% skim milk, were added for 2 h. The strips were washed and the reaction was developed with AEC (Pierce Inc, IL, USA). Samples were analyzed by ELISA and immunoblotting with polyclonal antisera. After washing, the plates were incubated with a 1 : 2,000 dilution of streptavidin (Sigma Chemical Co., St. Louis, MO, USA) at 100 *μ*L/well for 30 min at room temperature and washed 6 times. TMB (Sigma Chemical Co., St. Louis, MO, USA) in 30% H_2_O_2_ substrate was added and the color change reaction was stopped by the addition of 4 N H_2_SO_4_. Readings were taken at 450 nm using a microplate spectrophotometer (Multiskan MCC, Lab Systems and Flow Lab, Finland).

### 2.7. Cytotoxicity Assay

HeLa (CCL-2) and Vero (CCL-81) cells were used. The culture protocol recommended by the American Type Culture Collection (ATCC) was reproduced by the Adolfo Lutz Institute (cell culture collection), and cells were grown in Minimum Essential Medium Eagle (MEM). HeLa and Vero cells were plated at approximately 1.4 × 10^4^ cells/well on 96-well plates in MEM. Cytotoxic effects of the toxoids were visualized by neutral red (NR) vital staining containing antibiotics that were prepared one day before the assay and incubated overnight at 37°C. The medium containing the test compound was removed and replaced with 200 *μ*L of 25 *μ*g/mL neutral red diluted in MEM (NR medium without FBS) 3 h before termination of the experiment. After 3 hours, the lysosomes had taken up sufficient NR stain. The NR medium was then removed, the cells were rinsed twice with preheated PBS to remove excess unincorporated stain, and, after quickly rinsing in 100 *μ*L of fixative (0.5% formalin/PBS), 100 *μ*L of destain solution (1% glacial acetic acid, 49% PBS, and 50% ethanol) was added to each well to fix the cells and remove the NR in the solution.

The plates were gently shaken for 10 min on an orbital shaker and the absorbance of the solution was read at 550 nm. For the evaluation of cytotoxicity, the average absorbance of the media control well, which contained no chemical, was regarded as 100%, and the percentage of viable cells in each well was calculated according to (O.D. × 100)/control media, which is equivalent to the percentage of viable cells per well [28, 29]. The concentrations of cytokines were determined with reference to a standard curve for serial twofold dilutions of murine recombinant cytokines.

### 2.8. Specific Antibody Levels

Specific antibody levels (IgG, IgG1, IgG2a, IgG2b, IgM, and IgA) were measured by ELISA. Polystyrene plates (Maxi Sorp, NUNC, Denmark) were coated overnight at 37°C with 2 *μ*g/well OMP antigen in 0.5 M carbonate/bicarbonate buffer, pH 9.6, and washed with PBS-0.05% Tween, pH 7.4. After blocking for 1 h at 37°C in 5% skim milk (Molico, Nestlé) in PBS-0.05% Tween, pH 7.4, and after five washes, the samples were diluted in PBS-0.05% Tween plus 1% skim milk. Anti-IgG, -IgM, -IgA, -IgG1, -IgG2a, and -IgG2b conjugates (Kirkegaard and Perry, Gaithersburg, MD) were used as described previously and the reaction was developed for 20 min with a substrate consisting of TMB and 150 *μ*L and 30% hydrogen peroxide in 30 mL PBS, pH 7.4. The reaction was stopped by the addition of 4 N H_2_SO_4_. Readings were taken at 450 nm using a microplate spectrophotometer (Multiskan MCC, Labsystems and Flow Lab, Filand, USA).

### 2.9. Collection of Secretion and Serum

Serum samples were collected 28 days after i.n. immunization and 7 days after i.m. immunization. At 7 days after the last i.m. immunization, 100 *μ*L of saliva was collected from female mice injected i.p. with a single injection of 0.1 mg Pilocarpine HCL (Sigma Chemical Co., St Louis, Mo) in 100 *μ*L of PBS to stimulate salivation. Vaginal washes were taken from female mice by washing 3 times with 100 *μ*L of Dulbecco's medium using a pipettor fitted with a plastic tip [30]. Saliva was collected of the mice immediately after salivation that had been induced by a single intraperitoneal injection of 0.1 mg Pilocarpine HCL in 100 *μ*L of PBS. The samples were frozen at −20°C in 1.5-mL microcentrifuge tubes (BioRad) and subsequently extracted with 500 *μ*L of PBS containing 5% skim milk (Molico, Nestle do Brazil) and the protease inhibitor PMSF (Sigma Chemical Company, St. Louis, MO, USA). Conjugated goat anti-mouse antibodies (Kirkegaard and Perry, Gaithersburg, MD) were used as a conjugate.

### 2.10. SBA

The serum bactericidal activity (SBA) assay was performed with an agar overlay method in microtiter plates as described previously [31]. In brief, twofold dilutions of sera, starting at 1 : 2, were tested with a meningococcal inoculum of approximately 80 to 100 CFU per well of the homologous *N. meningitidis* strain, first grown overnight on brain heart infusion agar with 1% horse serum, and then grown for 4 h in a 5% CO_2_ atmosphere at 37°C on a new plate. Baby rabbit sera from an individual without bactericidal antibodies to the strain were used as a complement source. Agar was added to the plates after a 30-minute incubation of the reaction mixture at 37°C. The numbers of CFU were counted after overnight incubation in 5% CO_2_ at 37°C. The titers are given as the highest reciprocal final dilution of serum killing more than 50% of the inoculum.

### 2.11. Spleen Cell Proliferation Assay

Briefly, mice were sacrificed by cervical dislocation and the spleens were aseptically removed. Spleen cells were harvested by flushing the spleen with PBS. Red blood cells were eliminated by treatment with a hypertonic solution of NH_4_Cl for 3 min on ice followed by centrifugation and washing. Cells were stained with Trypan blue (Sigma) and resuspended at 8 × 10^6^ cells/mL in RPMI-1640 medium supplemented with L-glutamine, pyruvate, gentamicin, penicillin, and 10% FCS. Cells were cultured in 24-well plates (8 × 10^6^ cells/well, 2 mL/well). Culture supernatants were collected for cytokine determination.

### 2.12. Evaluation of Cytokine Production *In Vitro*

The cytokines IFN-*γ* (48, 72 h) and IL-6 (20, 48, 72 h) were measured in culture supernatants by capture ELISA using antibody pairs purchased from PharMingen (San Diego, CA) according to the manufacturer's instructions.

### 2.13. Statistical Analysis

One-way ANOVA statistical test was used to assess the significance of the differences between the various groups. In case of significant *F*-value, multiple comparison Tukey test was used to compare the means of different treatment groups and *P* < 0.05 was considered to be statistically significant.

## 3. Results

### 3.1. Electrophoretic Analysis

**Figure 1:**
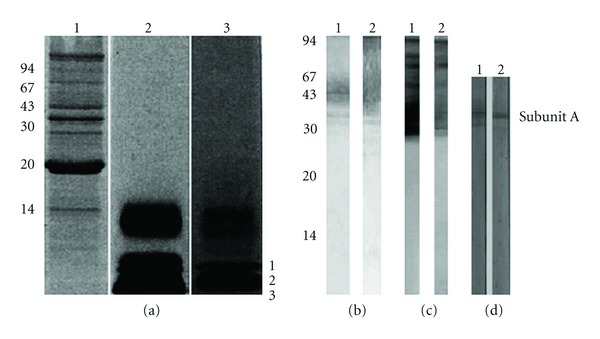
(a)(1) SDS-PAGE (13% gel) analysis of native OMPs of *N. meningitidis*; (a)(2) LPS characterization and (a)(3) LPS immunoreactivity with monoclonal antibodies of *N. meningitidis*; (b) *E. coli* stx1 (1) or stx2 (2) toxoid after silver stain. Standard markers are shown on the left of (a); (c) immunoblot of 13% SDS/PAGE and transfer to nitrocellulose membranes. Nitrocellulose membranes containing OMPs of *N. meningitidis* (lanes (c)(1) and (c)(2)) were incubated with 1 : 500 dilution of rabbit antiserum anti-OMPs-Stx1 or anti-OMPs-Stx2, respectively. Immunoblot with 100 *μ*g of toxoid Stx1 or Stx2; Lanes (d)(1) and (d)(2): IgG immunoreactivity of the monoclonal antibody anti-Stx1 or -Stx2, respectively, with the A subunit of the toxoid.

Figure 1 analyzed the OMP of *N. meningitidis* by SDS-PAGE with Coomassie blue and silver staining or monoclonal antibody reactivity to Stx1 or Stx2 toxins and toxoids of *E. coli*. SDS-PAGE after Coomassie staining showed the antigenic characterization of the *N. meningitidis *OMP. We observed proteins of 13 to 120 kDa, with major proteins of 46 kDa (class 1—PorA) and 38 kDa (class 3—PorB) proteins and a major protein of 28 kDa (class 5). The major OMPs of meningococcus have been classified on the basis of molecular weight and behavior on SDS-PAGE [32]. This antigen preparation was used for immunization Figure 1(a)1. For a better characterization of *N.* *meningitidis* strain used in this study the Figure 1(a)2 shows the antigenic characterization of *N. meningitidis* LPS after silver staining, and Figure 1(a)3 shows antigens lower than 10 kDa. Also Figure 1(a)3 shows immunoblot reacted with three different monoclonal antibodies. The bands at position 3.1 reacted with antibody WBE12-C10 L3,7,9, (5.9 kDa), 3.2 reacted with L1 (4.8 kDa) 3G3-1-8C, and 3.3 reacted with (3.6 KDa) L8, the 6E7-10 monoclonal antibody. The purified Stx1 and Stx2 toxoids and the native toxins of *E. coli *were also analyzed by SDS-PAGE in Figures 1(b)1 and 1(b)2 and in Figures 1(c)1 and 1(c)2, respectively. With SDS-PAGE, we can see a pattern of Stx1 and Stx2 toxins in Figures 1(b)1 and 1(b)2 and the concentrated toxoids Stx1 and Stx2 with peptides in the range of 30 to 100 kDa, respectively. The immunoblot (D 1,2) was reacted in sequence with two different monoclonal antibodies against the A subunit of Stx1 and Stx2, respectively.

### 3.2. Cytotoxicity

Stx1 and Stx2 toxoids of *E.coli* strains used were consequently tested for cytotoxicity using the cultured supernatants of Vero and HeLa cells. We have used a quantitative colorimetric method based on the uptake of NR dye, which accumulates in the lysosomes of uninjured cells. After detoxification with formaldehyde, neither stx1 nor stx2 was cytotoxic to Vero or HeLa cells (data not shown) [28, 29].

### 3.3. Detection of IgG, IgM, and IgA in the Sera from Mice by ELISA

**Figure 2:**
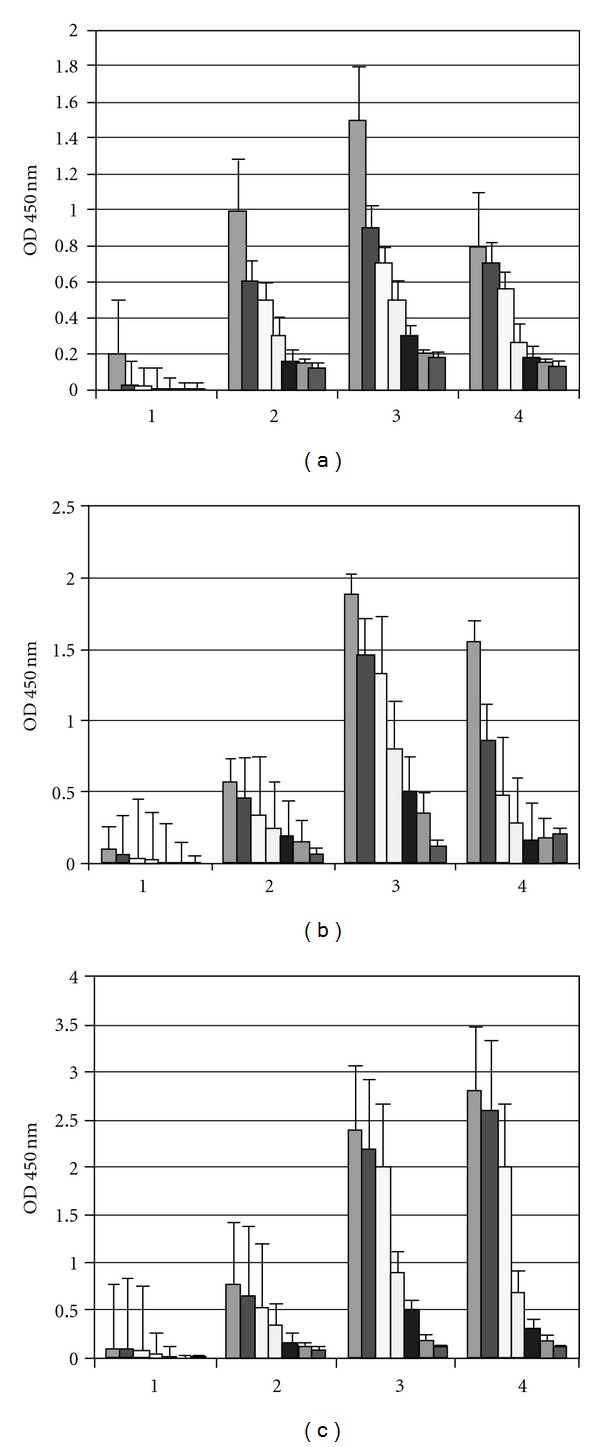
Antigen-specific IgG, IgM, and IgA antibody responses in mice sera after i.n. immunization with native OMPs of *N. meningitidis* determined by ELISA at 28 days with Stx1, Stx2, or CT as adjuvants. Results are shown as mean titers and error bars indicate standard deviations from the mean. (a) Sera pooled from nine mice immunized with OMPs and stx1; (b) sera pooled from eight mice immunized with OMPs and stx2; (c) sera pooled from ten mice immunized with OMPs and CT. (1) IgG, IgM, and IGA of normal mice; (2) IgG; (3) IgM; (4) IgA. Each scale bar represents the mean ± SE of nine to ten mice per group in an experiment representative of two separate experiments. (a) OMPs and Stx1- IgG, IgM, and IgA containing formulations versus normal sera (*P* < 0.05); (b) OMPs and Stx2 IgG, IgM, and IgA containing formulations versus normal sera (*P* < 0.001); (c) OMPs and CT IgG, IgM, and IgA containing formulations versus normal sera (*P* < 0.05).

We evaluated the immunogenicity of the OMP from *N. meningitidis *following i.n. delivery with Stx1, Stx2 or CT as an adjuvant in neonatal mice (Figures 2(a)–2(c)) and i.n./i.m. immunization in adult mice that induced high titers of specific antibodies. We verified that the OMPs of *N. meningitidis* induced antibody titers in adult BALB/c mice corresponding to the toxoids used. To better interpret these results, we used the ratio between the values of the postimmunization serum samples and the value of the preimmune sera compared with the normal sera. Increases in the antibody titers suggested that four doses of OMP in mice in neonates successfully primed the immune system of mice to generate fast IgG, IgM, and IgA antibody production. We observed a fast production of antibodies 7 days after the boost. 

### 3.4. The Prime-Boost Immunization Protocol Induces a Th1-Biased Response

To evaluate the effect of the vaccination strategy on the elicited IgG response, we analyzed the predominance of the IgG1, IgG2a, or IgG2b isotypes, Figure 5—representative of Th2- and Th1-type immunity, respectively—within the pool of specific serum antibodies using OMP Stx1 or Stx2 toxoids or CT. As previously reported by our group, when the prime-boost schedule was used with other antigens, such as NOMV of *N. lactamica* in neonatal [27] or adult mice [33] with a purified protein of *N. meningitidis*, an immune response could be induced quickly. 

### 3.5. Mucosal Immune Response

**Figure 3:**
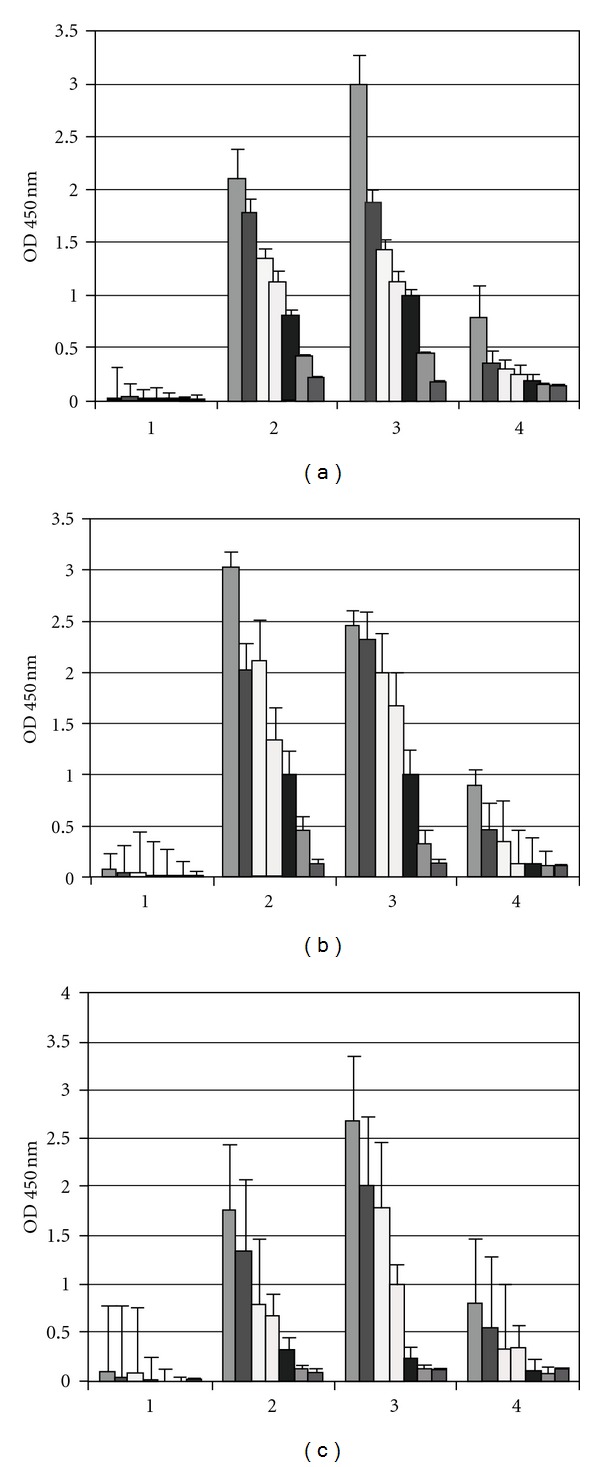
Antigen-specific IgG, IgM, and IgA antibody responses in serum stimulated after i.n./i.m. immunization with native OMPs of *N. meningitidis* at 42 days, 7 days post the i.m prime-boost, in the presence of stx1, stx2, or CT as adjuvants. (b) Sera pooled from nine mice immunized with OMPs and stx1; (a) sera pooled from eight mice immunized with OMPs and stx2; (c) sera pooled from ten mice immunized with OMPs and CT. (1) IgG, IgM, and IgA of normal mice; (2) IgG; (3) IgM; (4) IgA. Each scale bar represents the mean ± SE of nine to ten mice per group in an experiment representative of two separate experiments. (a) OMPs and stx1- IgG, IgM, and IgA containing formulations versus normal sera (*P* < 0.001), (b) OMPs and stx2 IgG, IgM, and IgA containing formulations versus normal sera (*P* < 0.001); (c) OMPs and CT IgG, IgM, and IgA containing formulations versus normal sera (*P* < 0.05).

**Figure 4:**
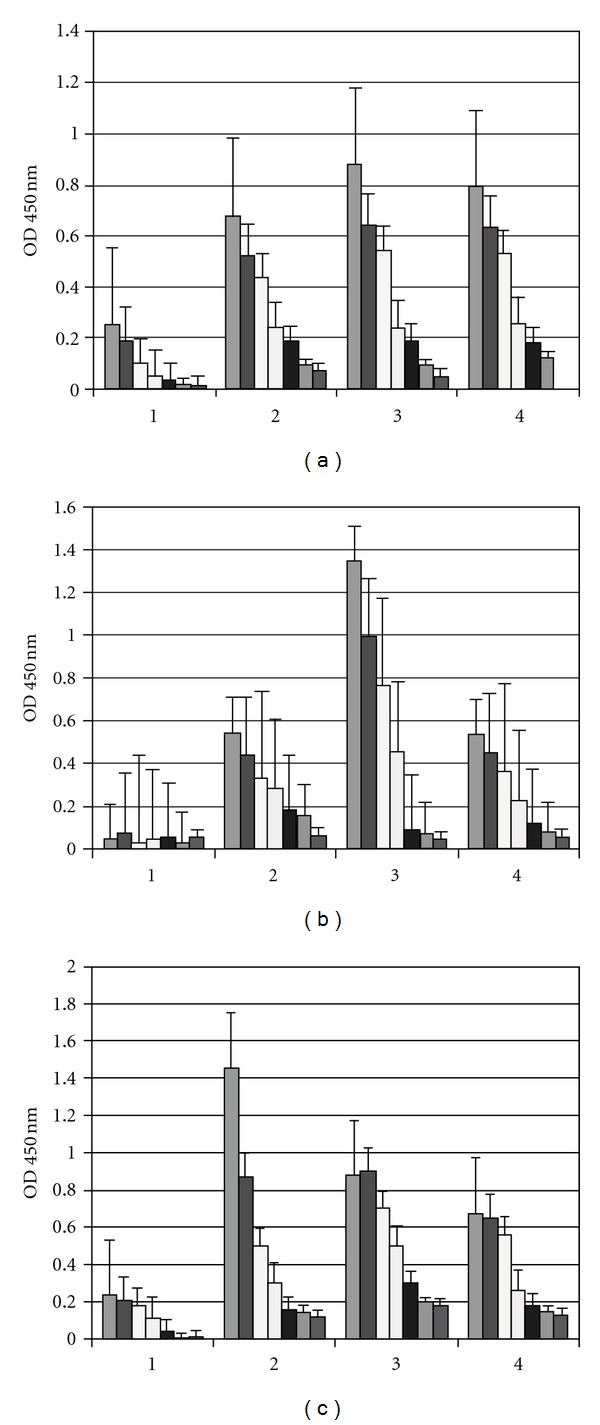
Concentration of specific anti-OMPs IgA in saliva and vaginal washes of adult mice immunized i.n./i.m. at 42 days, 7 days post the i.m. prime-boost. (a) IgA saliva. (1) IgA saliva antibodies in normal mice; (2) IgA saliva antibodies pooled from nine mice immunized with OMPs and Stx1; (3) IgA saliva antibodies pooled from eight mice immunized with OMPs and Stx2; (4) IgA saliva antibodies pooled from ten mice immunized with OMPs and CT (b) IgA of vaginal washes. (1) IgA vaginal washes antibodies in normal mice; (2) IgA vaginal washes antibodies pooled from nine mice immunized with OMPs and Stx1; (3) IgA vaginal washes antibodies pooled from eight mice immunized with OMPs and Stx2; (4) IgA vaginal washes antibodies pooled from ten mice immunized with OMPs and CT. Each scale bar represents the mean ± SE of mice per group in an experiment representative of two separate experiments. (a) OMPs and stx1 IgA containing formulations saliva versus vaginal washes (*P* < 0.001). (b) OMPs and stx2 IgA containing formulations saliva versus vaginal washes (*P* < 0.001). (c) OMPs and CT IgA containing formulations salivaversus vaginal washes (*P* < 0.05).

**Figure 5:**
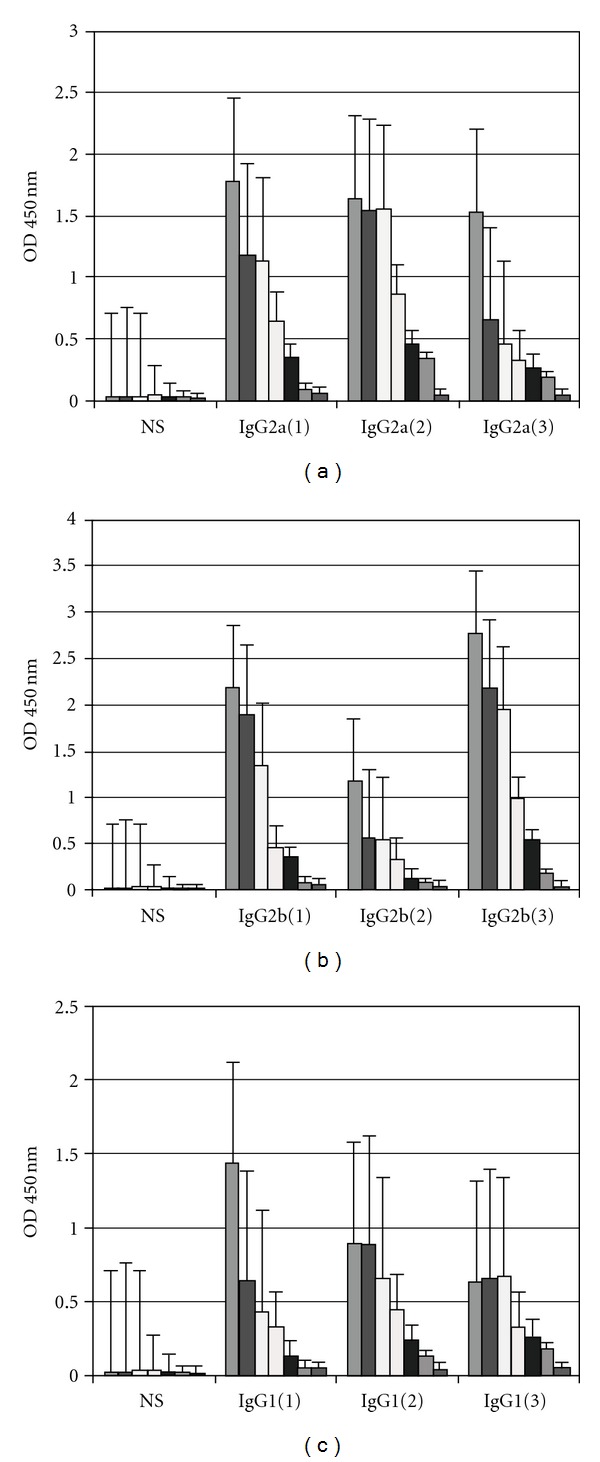
Level of IgG1, IgG2a, and IgG2b antibodies present, determined by ELISA, in mouse sera of BALB/c immunized with (a) OMPs of *N. meningitidis* and stx1 (b) OMPs of *N. meningitidis* and stx2 and (c) CT, at 42 days, 7 days post the i.m prime-boost. NS: normal mouse sera as control. Each scale bar represents the mean ± SE of nine to ten mice per group per group in an experiment representative of two separate. (a) OMPs and stx1-IgG1, IgG2a, and IgG2b containing formulations versus normal sera (*P* < 0.001). (b) OMPs and stx2 IgG1, IgG2a, and IgG2b containing formulations versus normal sera (*P* < 0.001). (c) OMPs and CT IgG1, IgG2a, and IgG2b containing formulations versus normal sera (*P* < 0.05).

We analyzed the level of IgA produced with the new toxoids compared with CT in nasal and vaginal fluids after immunization Figure 4. The formulations induced a good immune response on the 42th day after immunization at the mucosal level in the groups of mice primed during the neonatal period (Figure 3(a)). In addition, the IgA antibodies could be compared with the vaginal secretions (Figure 3(b)). Differences in salivary IgA anti-OMP responses were observed on the 42th day after immunization (Figure 3(a)). However, no significant differences were detected between groups using the new toxoids compared with the classical mucosal adjuvant CT in i.n./i.m-immunized groups compared to saliva and intravaginal secretion (Figure 3(c)). The IgA responses in mice immunized by the i.n. route with the antigen preparations persisted for 8 weeks following initial immunization in nasal and vaginal wash secretions (data not shown). 

### 3.6. Immunoblot Reactivity with Polyclonal Antibodies

**Figure 6:**
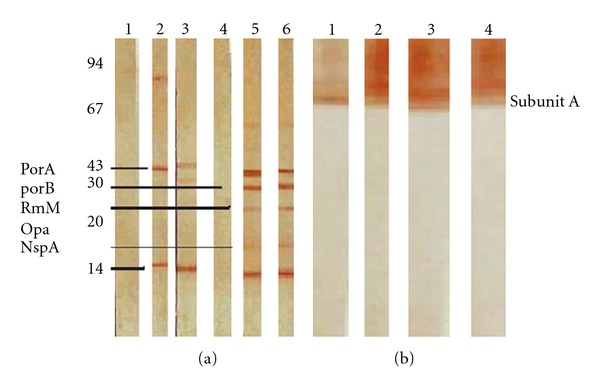
Immunoblot reactivity showing IgG antibodies binding to OMPs of *N. meningitidis*. (a)(1)–(6) With OMPs of *N. meningitidis* (B: 4:P1.15,19,5.5,L379,1,8) 28 days after i.n. immunization in the presence of stx1 (a)(1) or stx2 (a)(2). Immunization-specific IgG antibody responses at 42 days, 7 days post the i.m prime-boost in the presence of stx1 (a)(5) and stx2 (a)(6) as adjuvants in serum. Immunoreactivity of normal sera used as a control in (1,4). In (b)(1,2), immunoblot with sera of BALB/c mice immunized with OMPs and toxoids. IgG reactivity with (1) OMPs and stx1 28 days after i.n. immunization (2) OMP and stx1 42 days, 7 days post the i.m prime-boost and (b)(3,4) immunoblot with sera of BALB/c mice immunized with OMPs and toxoids. IgG reactivity with (1) OMPs and stx2 28 days after i.n. immunization (2) OMPs and stx2 42 days, 7 days post the i.m prime-boost. Molecular weight marker is on the left of (a).

As shown in Figure 6(a), we observed immunoreactivity present in the IgG antibodies from the sera of mice after i.n. and i.n./i.m. in a prime-boost immunization using different toxoids against OMP of *N. meningitidis*. The main antigens recognized by IgG antibodies were PorA (class 1), PorB (class 2), RmpM (class 4), Opa (class 5), and Neisserial surface protein A (NspA). As shown in Figure 6(b), we also observed immunoreactivity of IgG antibodies against the A subunits of Stx1 and Stx2 toxoids. The molecular position of the A subunit changed, which could have been attributed to the treatment used in toxoid preparation. In Figure 6(a)(2), we saw IgG reactivity with OMP and Stx1 at 28 days after i.n. immunization and in Figure 6(a)(3) we saw IgG reactivity with OMP and Stx1 at 42 days, 7 days post the i.m. prime-boost. In Figure 6(a)(5), we saw IgG reactivity with OMP and Stx2 28 days after i.n. immunization and in Figure 6(a)(6) with OMP and Stx2 42 days, 7 days post the i.m. prime-boost. In Figure 6(a)(1) we saw normal sera of neonate mice 12 days and 6(a)(2) normal sera of adult mice. In Figure 6(b)(1), we saw IgG reactivity with Stx1 at 28 days after i.n. immunization and in Figure 6(b)(2) IgG reactivity *i*th Stx1 at 42 days, 7 days post the i.m. prime-boost. In Figure 6(b)(3), we saw IgG reactivity with Stx2 28 days after i.n. immunization and in Figure 6(b)(4) Stx2 42 days, 7 days post the i.m. prime-boost.

### 3.7. Determination of the Cytokines in BALB/c Mice

**Figure 7:**
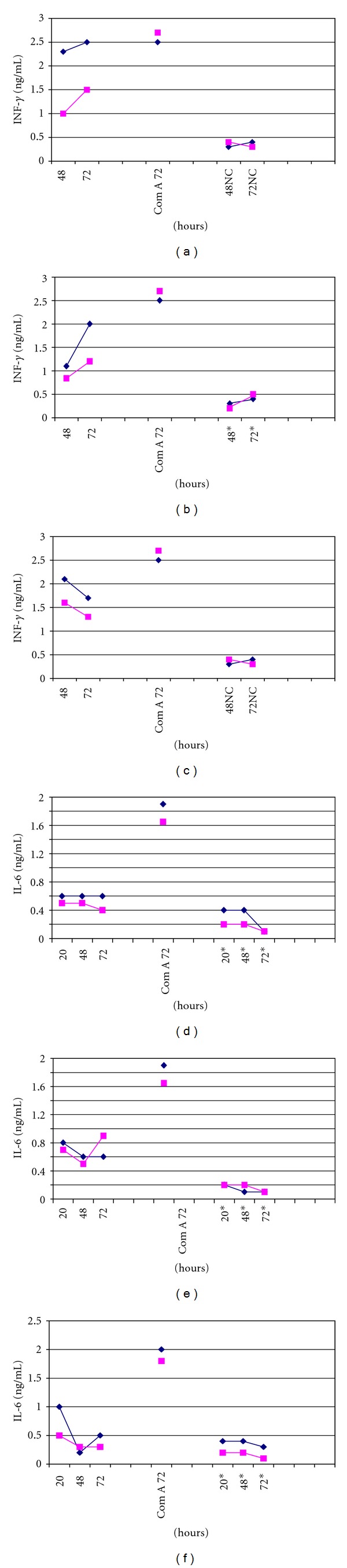
Production of IFN*γ* and IL-6 in adult BALB/c mice after four i.n. doses of 20 *μ*g of OMP of *N. meningitidis* and 2 *μ*g of (a) Stx1 toxoid, (b) Stx2 toxoid, or (c) CT. The spleen cultures were stimulated *in vitro* with 1 or 2 *μ*g of the homologous antigen used for immunization, and the supernatants were harvested after 48 h, and 72 h of culture for IFN-*γ* or 20 h, 48 h and 72 h for IL-6. IFN-*γ* concentrations are expressed in ng/mL. IL-6 concentrations are expressed in pg/mL. Normal cells* and concanavalin A were used as controls.

To examine whether type 1 (IFN-*γ*) and type 2 (IL-6) cytokine profiles were differentially secreted in BALB/c mice, spleen cell supernatants were analysed 20, 48 and 72 h after i.m. immunization with OMP-Stx1 or -Stx2 in order to determine the presence of cytokines by capture ELISA. We used 1 or 2 *μ*g of OMP. As shown in Figure 7, BALB/c mice immunized with OMP of *N. meningitidis *and Stxs showed mixed type 1 and type 2 cytokine responses. Comparing cytokine levels present in the control mice, we could verify that, despite the Stx1 or Stx2 toxoids used, high levels of type 1 and type 2 cytokines were produced independently of the dose used. Moreover, all i.n.-vaccinated groups showed higher IFN-*γ* production when compared to unimmunized mice. IFN-*γ* mediates diverse functions in bone marrow-derived phagocytes, including phagocytosis and microbe destruction. This cytokine has also been detected at implantation sites under both physiological and pathological conditions in many different species. Cytokines IFN-*γ* and IL-6 were produced by spleen cells upon *in vitro* recall with the OMP of *N. meningitidis* and were detected by specific *in vitro* assays.

### 3.8. Bactericidal Activity

The bactericidal activity of each serum was assessed against the meningococcal homologous strain B:4:P1.15,19,5.5,L379,1,8. Bactericidal activity was assayed relatively with the antibodies raised against OMP of *N. meningitidis*.  Table 1 shows the bactericidal titers of the antibodies. Sera collected after i.n. immunization showed lower antibody titers compared to the i.m. immunization. We observed a significant difference between the bactericidal antibody response after the i.m. dose compared with the i.n dose. These data suggest that high levels of bactericidal antibodies were produced that are specific for OMP. The controls showed a lower bactericidal activity than that observed in the experimental groups.

### 3.9. IgG and IgM Avidity

To investigate the type of antibody response produced, we determined the IgG and IgM avidity index (AI) after i.n. and i.m. immunization in all of the studied groups. We found that the AI of the IgM and IgG isotypes did not differ significantly between the different adjuvants used. Additionally, we observed a good correlation between AI and bactericidal activity after i.n. or i.m. immunization. Interestingly, we also observed a good correlation between the bactericidal activity of postimmunization sera samples and the antibodies produced against OMP. Our data suggest that use of these adjuvants resulted in the production IgG and IgM antibodies of high avidity (Table 2). The AI was considered during the analysis of the results as follows: AI values less than 0.29 were designated as low avidity, 0.30–0.49 were designated as intermediate avidity, and greater than 0.50 were high avidity.

## 4. Discussion

This study demonstrated that Stx1 and Stx2 toxoid-based vaccines combined with OMP of *N. meningitidis* B resulted in a dramatic improvement in the use of Stx1 and Stx2 as toxoid delivery systems as adjuvants, with the production of antibodies of high avidity and bactericidal activity against *N. meningitidis* B in neonatal mice using a prime-boost immunization schedule. This vaccine formulation was also efficient in inducing the production of IFN-*γ* and IL-6. Specifically, following i.n./i.m. immunization of mice with vaccine preparations compared with OMP plus the cholera toxin B subunit, we also observed increased production of IgG2a/2b versus IgG1 Abs, as well as IFN-*γ*, indicating the induction of a Th1 response. Thus, these data indicate a mixed Th1/Th2 immune response. This observation also has important ramifications for vaccine development against meningococcus. The results demonstrate that Stx1 and Stx2 induced specific antibody responses with bactericidal activity. The neutralization of toxicity occurred without compromising immunogenicity.

In practice, however, designing and producing affordable new vaccines against existing pathogens is time consuming, expensive, and without guarantee of success. The ability to elicit vaccine antigen-specific immune responses of the appropriate type and magnitude is a key issue in vaccinology. This issue is compounded somewhat by the propensity of neonates and adults to respond differently to the same vaccine preparations [34].

We know that there is a great need for immunological adjuvants or vectors that are capable of stimulating both antibody and cytolytic T lymphocyte responses to vaccine antigens. An interesting work showed that StxB of *S. dysenteriae* was a powerful vaccine delivery system for polyepitopic antigens that can elicit antigen-specific CTLs, humoral immune responses, and Th1 polarization without the use of adjuvants to tumor or viral infection [35, 36].

As described previously, the choice of administration route may be very important for effective induction of mucosal immune responses. Mucosal vaccines delivered into the nasal tract provide several advantages. Intranasal immunization effectively induces not only systemic IgG but also secretory IgA responses in mucosal effector tissues. In this study, i.n. immunization induced higher levels of secretory IgA and IgM in secretions than systemic IgG, IgM, and IgA. In addition, i.n. immunization may involve less of a risk of anaphylactic reactions, since the doses used in i.n. immunization induce lower total and antigen-specific IgE levels in serum [37, 38]. Another point that will be important to analyze in future studies is the mechanism(s) by which these toxoids stimulate the mucosal immune responses, as this has remained largely obscure to date. Thus, intranasal vaccination using the new toxoids as adjuvants may be useful in meningococcal human disease prevention, although it remains to be determined whether the antibody responses elicited in humans will be sufficient to confer protection.

As we know, the design of protein-based meningococcal vaccines is complicated by the high level of genetic and antigenic diversity of meningococcus. Because it is naturally competent for genetic transformation and recombination, meningococcus has a complex population structure reflecting the combined impact of accumulated mutations and horizontal genetic exchange [39].

In a recent interesting review, Feavers and Pizza [39] presented a comparison with the development and implementation of other vaccines against bacterial diseases of the young and highlighted the slow progress toward the prevention of group B meningococcal disease through vaccination.

Only OMV vaccines have been used, ostensibly to disrupt clonal outbreaks, but because of the restricted range of protection they offer against diverse hypervirulent lineages, their use in routine immunization programs has been limited. Emerging evidence suggests that meningococcal component vaccine formulations are likely to consist of multiple antigens [39].

 In the case of abundant immunodominant antigens, which tend to be antigenically variable, vaccines are likely to consist of a number of antigenic variants so that they offer the required breadth of coverage against diverse meningococcal isolates [40]. In the case of the less abundant and less variable antigens, vaccines are likely to consist of multiple components to take advantage of synergistic effects. New meningococcal vaccine formulations may also include novel adjuvants to ensure potent bactericidal antibody responses [27, 33].

How relevant these observations are to protection needs more studies. Meanwhile, we observed the production of antibodies with bactericidal activity and high avidity independent of the toxoid used. As we know, the heat-labile enterotoxins have attracted considerable attention due to their exceptional mucosal adjuvant properties, although their intrinsic enterotoxicity precludes their use as adjuvants for human vaccines [41].

In this study, the priming effect of OMP and Stx1 or Stx2 used as adjuvants did not seem to be similar in intensity and quality to antibodies produced for OMP antigens. The same seemed to be true in the induction of IFN-*γ* and IL-6 when Stx1 or Stx2 was used as adjuvants. These results suggest that Stxs can be safely used as a priming stimulus in neonatal animals in a prime-boost strategy to control meningococcal infection. Here, for the first time, Stx1 or Stx2 was shown to be effective and safe mucosal adjuvants for the development of a nasal meningococcal immune response. The altered toxicity of Stx1 or Stx2 toxin might be closely related to a potent adjuvant action with antibody responses to OMP antigens of *N. meningitidis* B.

These data suggest the possibility of intranasal immunization with meningococcal antigens and toxoids Stx1 and Stx2 adjuvants as a new strategy in mucosal immunization. Another important point is that to what extent such dichotomy between localization of Th1 and Th2 cells occurs in mucosal inductive and effector tissues that remains to be determined.

Another important point in our studies was the antigen specificity of the mouse IgG response to OMP of *N. meningitidis* using Stx1 and Stx2 by immunoblotting. The immune responses were principally directed to class 1(PorA), class 2 (PorB), and Opa class 5 proteins of *N. meningitidis*. It was particularly noteworthy that mice that responded with high ELISA titers had high antibody avidity levels. The subclass response was to IgG1, IgG2a, and IgG2b. The class 1 protein is, therefore, an important antigen for inclusion in an OMP vaccine for several studies described in the literature.

Our studies using detoxified subunits of Stx1 or Stx2 agreed with the results of Ågren et al. [42] and indicated that the neutralization of the cytotoxicity was mainly due to the A subunit. On the other hand, an association between the cytotoxicity of the protein and its ability to induce cytokine release has already been suggested [43].

However, application of the toxoids or their subunits as adjuvants for human use requires an understanding of their mode of action and the separation of their desirable immunomodulatory properties from their toxicity [44]. It has been shown that the adjuvant action is not critically dependent upon the enzymatic activity of the A subunit, and that the isolated B subunit may exert different effects on cells of the immune system than do the intact toxins [45]. However, immunomodulatory effects of the enterotoxin and its subunits can result in the enhancement of immune responses and thus can be considered as immunoregulatory agents [45]. Immunomodulatory mechanisms mediated by each subunit of the holotoxin give a better understanding of the toxin's effect on the immune system and for application of these subunits as vaccine candidates, either as an adjuvant or as a vector [35].

Although characterizing the mechanisms involved in regulating mucosal immune responses has proven difficult, the availability of defined molecular probes, including enterotoxin derivatives, has provided new opportunities for research in this field of investigation [35, 46].

The bacterium O157:H7 is known to be the most important STEC serotype in many industrialized countries, hundreds of distinct STEC serotypes have been isolated from human diseases in many geographic areas, including Brazil, and, of the 20 distinct serotypes identified, more than 50% corresponded to serotypes associated with human diseases [47]. As we know, the outer membrane of meningococcus is considered a good adjuvant [48]. As our preliminary results and previous studies have shown, antibodies protect against STEC infection (data not shown). Additionally, studies are under way in our laboratory using other animal models, including adult mice and rabbits.

As we know, the major enterotoxins produced by *V. cholerae* and *E. coli*, CT and LT, respectively, have continued to be the most studied mucosal adjuvants [36]. The B subunit of molecules binds to cell surface gangliosides and this could enhance immune response by increasing the permeability of epithelial membranes.

As the major enterotoxins studied in the literature, with this new toxoids of STEC *E.coli* we hoped to open the opportunity to introduce new adjuvants, especially for *N. meningitidis* B. As recently pointed out by two experts in the area of meningococcus, when we compare the development and implementation of other vaccines against bacterial diseases of the young, progress towards the prevention of group B meningococcal disease through vaccination it has been considered slow. Only OMV vaccines have been used, ostensibly to disrupt clonal outbreaks, but because of the restricted range of protection they offer against diverse hypervirulent lineages, their use in routine immunization programs has been limited. In addition to the study of new meningococcal vaccine formulations, they also emphasize the use of novel adjuvants to ensure potent bactericidal antibody responses.

Finally, our results demonstrated an interesting result that OMP and the toxoids Stx1 or stx2 used as adjuvants were recognized by immunoblot at 28 days, after a prime, and at 42 days after the prime-boost schedule used in the present investigation, the neisserial surface Porin A and (NspA) was first identified using a monoclonal antibody and is a highly conserved, and surface-exposed outer membrane protein of *N. meningitidis* has been shown to induce a bactericidal immune response in animals against all pathogenic Neisserial serogroups obtained following the immunization of mice with an outer membrane preparation [49]. It has a conserved primary structure, is expressed by most meningococci, and elicits antibodies with bactericidal activity against diverse meningococcal isolates in mice. More studies are necessary in order to understand the importance of this antigen at a mucosal level. Additionally, the immunization schedule in the present investigation produced IgG antibodies that recognized PorA, PorB, RmM, and OpA, studied for their importance in several vaccines used until this time, especially for *N. meningitidis* B [49].

As we know, since the publication of the entire genome of *N. meningitidis* at the beginning of this decade, several studies focusing on alternative antigens to compose a new vaccine against *N. meningitidis* serogroup B (Men B) have been described [40]. Meanwhile, we do not have a good vaccine that gives a good protection in all ages. The degree of strain- and serosubtype-specific responses varies with age, so only OMV vaccines have been used, ostensibly [50].

The discovery of new adjuvants, as in our study, represents a major step in our understanding of a new protective vaccine that induces responses at mucosal surfaces. However, there are still many unanswered questions, such as how the mechanisms operate at the mucosal level as well as several adjuvants described in the literature. In addition, we know that we have different types/strains of meningococcus B bacteria, so a universal vaccine against all types is a goal. A great deal of work needs to be done on the precise regulatory mechanisms that govern the efficient generation of new vaccines against this pathogen.

## Figures and Tables

**Table 1:** SBA in groups of mice after prime i.n. and boost i.m. with meningococcal OMP+Stx1 or OMP+Stx2 or OMP+CT.

Routes	Unimmunized mice*		OMP+Stx1		OMP+Stx2		OMP+CT	
Intranasally	4	—	16	—	8	—	4	—
Intramuscularly	—	2	—	164	—	512	—	128

*Normal serum from neonatal unimmunized mice or adult mice.

**Table 2:** Avidity index of IgG and IgM antibodies of immunized mice by intranasally and intramuscularly routes.

Routes	Unimmunized mice*		OMP+Stx1		OMP+Stx2		OMP+CT	
	IgG	IgM	IgG	IgM	IgG	IgM	IgG	IgM
Intranasally	0.16	0.13	0.82	0.65	0.94	0.78	0.42	0.35
Intramuscularly	0.12	0.13	0.45	0.69	0.84	0.65	0.33	0.38

Avidity index values: <0.29: low avidity; 0.30–0.49: intermediate avidity; >0.50: high avidity.
